# Immunosenescence in prostate cancer: from aging-related immune dysfunction to therapeutic opportunities

**DOI:** 10.3389/fimmu.2026.1852294

**Published:** 2026-07-23

**Authors:** Juntao Guo, Ke Wu, Zheng Ma, Shuai Guo, Fei Wang, Lingxiang Lu

**Affiliations:** 1 Department of Urology, Suzhou Ninth People’s Hospital, Soochow University, Suzhou, Jiangsu, China; 2 Department of Urology, The Affiliated Suzhou Hospital of Nanjing Medical University, Suzhou Municipal Hospital, Gusu School, Nanjing Medical University, Suzhou, Jiangsu, China

**Keywords:** aging, cellular senescence, immunosenescence, immunotherapy, inflammaging, prostate cancer, SASP, tumor microenvironment

## Abstract

Prostate cancer is a typical age-associated malignancy, and increasing evidence suggests that age-related immune alterations play an important role in its initiation, progression, and therapeutic response. Immunosenescence, characterized by impaired immune surveillance, reduced effector-cell function, contraction of the naïve T-cell repertoire, and persistent low-grade inflammation, may contribute to the establishment of a tumor-permissive microenvironment in older patients. In prostate cancer, these changes are particularly relevant because the disease often develops in an immunologically “cold” and suppressive tumor milieu. In addition to systemic immune aging, cellular senescence and the senescence-associated secretory phenotype (SASP) further reshape the local microenvironment by promoting chronic inflammation, stromal remodeling, and immune dysfunction. Together, immunosenescence, inflammaging, and senescence-related signaling may facilitate tumor persistence, immune escape, and resistance to therapy. Emerging therapeutic strategies therefore extend beyond tumor-intrinsic targets and include optimization of immunotherapy, reprogramming of the tumor microenvironment, and targeting of senescent cells or SASP-related pathways. A deeper understanding of how aging-related immune dysfunction interacts with prostate cancer biology may help improve patient stratification and support the development of more individualized treatment strategies for older prostate cancer patients.

## 1 Introduction

Prostate cancer is one of the most frequently diagnosed malignant tumors in men, and it is also a typical age-related disease. Its incidence and mortality increase significantly with age (1, 2). Although the accumulation of genetic and epigenetic alterations has long been considered as a core driver of prostate tumorigenesis, increasing evidence suggests that age-related changes in the immune system also play a key role in shaping the onset, progression, and treatment response of the disease (3, 4). Aging is accompanied by a progressive decline in immune capacity. This process is often called immunosenescence, which is characterized by reduced immune surveillance capacity, impaired effector cell function, contraction of the naive T-cell repertoire, and persistent low-grade inflammation (4, 5). In the context of cancer, these changes may not only reflect the decline of immune function, but also represent an immune disorder state, which can promote the persistence of tumor, immune escape and treatment resistance (5, 6).

In prostate cancer, the relevance of immunosenescence is particularly important because the disease predominantly affects older men and is often associated with an immunologically “cold” tumor microenvironment (2, 6). Unlike immunologically ‘hot’ tumors that contain abundant cytotoxic lymphocyte infiltration, prostate tumors frequently show limited effector T-cell activity, low antigen-presenting capacity, increased myeloid and regulatory immune components, and stromal barriers that restrict productive antitumor immunity (6, 7). Aging-associated immune dysfunction may reinforce these features by reducing naïve T-cell diversity, weakening CD8^+^ T-cell and natural killer cell cytotoxicity, impairing dendritic-cell priming, and promoting chronic inflammatory and myeloid-skewed immune remodeling (7, 8). Therefore, immunosenescence is not merely a general background feature of cancer in older individuals, but may directly contribute to several prostate cancer-specific biological characteristics, including immune exclusion, suppressive myeloid infiltration, stromal remodeling, androgen receptor–linked inflammatory signaling, disease persistence, and variable responses to therapy. In this mini-review, we summarize the major features of immunosenescence that are particularly relevant to prostate cancer, discuss how these changes shape the prostate tumor microenvironment and disease progression, and highlight therapeutic opportunities that may emerge from targeting aging-related immune dysfunction (5, 8).

## 2 Hallmarks of immunosenescence relevant to prostate cancer

Immunosenescence is a multifaceted process involving both adaptive and innate immune compartments, and its consequences extend beyond simple immune decline (9, 10). In the adaptive immune system, aging is associated with thymic involution, a progressive reduction in naïve T-cell output, and the accumulation of memory and terminally differentiated T cells with limited proliferative capacity (10, 11). As a result, the T-cell receptor repertoire becomes less diverse, reducing the ability of the immune system to recognize and respond effectively to novel antigens, including tumor-associated antigens (12, 13). In parallel, aged T cells often exhibit functional exhaustion-like features, including decreased cytotoxicity, impaired cytokine production, and increased expression of inhibitory receptors, all of which can weaken antitumor immune surveillance (11, 13). Regulatory T-cell expansion has also been reported in aging-associated immune remodeling, further contributing to an immunosuppressive environment that may hinder effective tumor control (13, 14). B-cell compartments are likewise altered with age, with reduced generation of naïve B cells, impaired class switching, and less effective antibody responses, all of which contribute to broader immune dysfunction in older individuals (10, 14).

Aging also remodels innate immunity in a manner highly associated with tumorigenesis. Although the number of natural killer cells is usually maintained, their cytotoxic activity and the ability to clear malignant cells often decline (15, 16). Dendritic cells may exhibit defective antigen presentation, altered migration ability, and reduced ability to induce effective T cell responses, thereby weakening the link between innate sensing and adaptive antitumor immunity (17, 18). In addition, macrophages and neutrophils undergo functional reprogramming with age, usually presenting a phenotype characterized by enhanced chronic inflammatory signals but insufficient immune defense efficacy (18, 19). These changes may promote the production of immunosuppressive mediators, weaken phagocytosis, and support a tissue environment conducive to tumor growth (18, 19). Therefore, aging can cause a paradoxical state: immune cells are chronically activated for a long time, but their ability to initiate a coordinated antitumor response is decreased (9, 19).

A core feature connecting these alterations is inflammatory aging, a chronic, low-grade inflammatory state marked by elevated levels of cytokines such as interleukin-6, tumor necrosis factor-α, and interleukin-1β (9, 20). Rather than enhancing host protection, this persistent inflammatory milieu can promote genomic instability, stromal remodeling, immune suppression, and tumor-supportive signaling (20, 21).

In prostate cancer, where tumorigenesis and progression occur predominantly in older men, these age-related immune changes are likely to be especially relevant. Immunosenescence should therefore be viewed as a state of immune dysregulation in which reduced immune competence coexists with chronic inflammation, together creating conditions that may favor tumor establishment, immune escape, and disease progression (13, 21).

**Figure 1:**
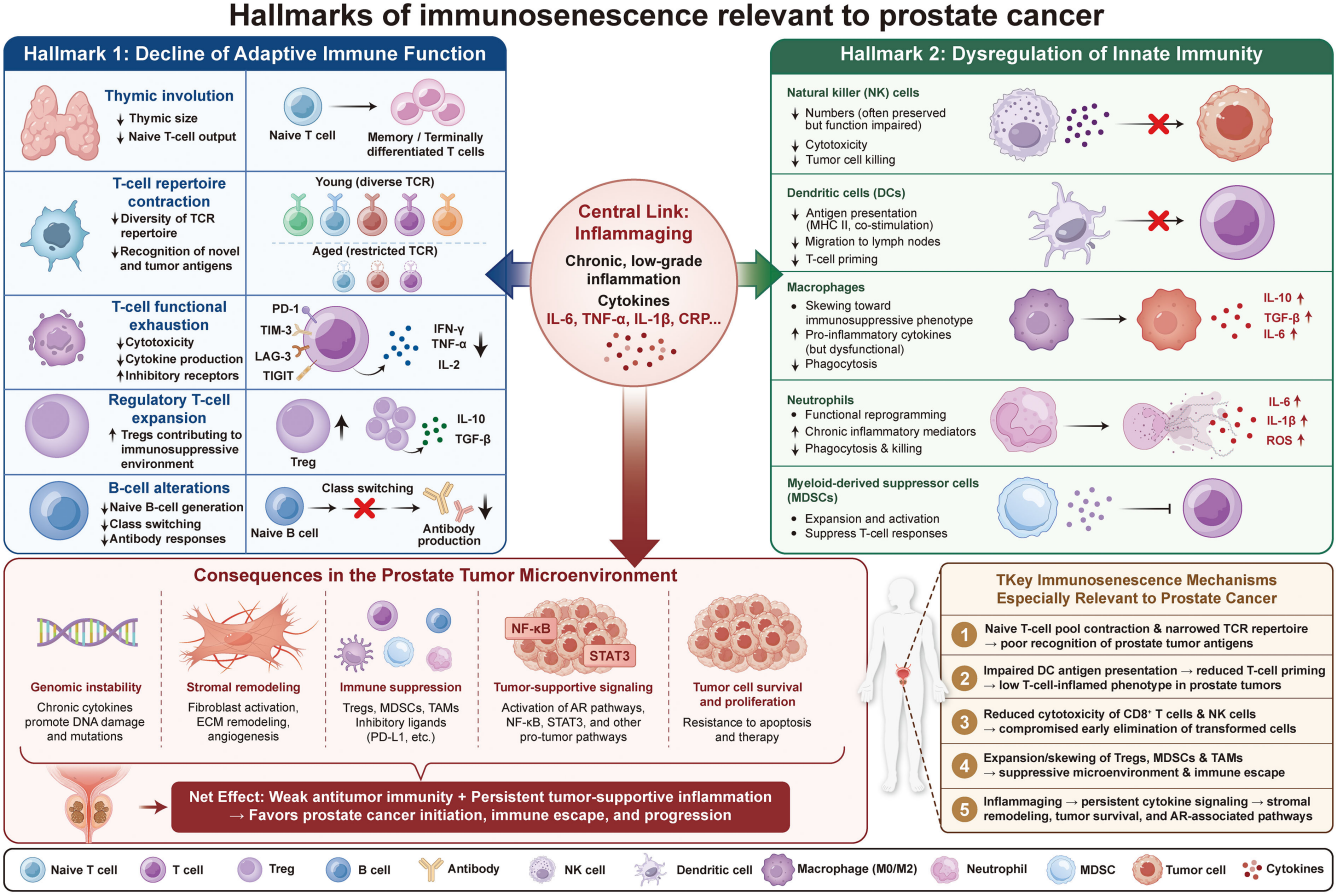
Integrative mechanism of immunosenescence and its consequences on antitumor immune surveillance.

Among the various characteristics of immune aging, several aspects seem to be particularly relevant to the biology of prostate cancer. First, the contraction of the naive T-cell pool and the narrowing of the T-cell receptor repertoire may limit the host recognition of prostate tumor-associated antigens, resulting in the weakening of adaptive immune surveillance (22, 23). Secondly, age-related impaired antigen-presenting function of dendritic cells may further reduce the T-cell priming effect and strengthen the common low T-cell-inflamed phenotype in prostate tumors (24, 25). Third, reduced cytotoxic activity of CD8^+^ T cells and natural killer cells may compromise early elimination of transformed prostate epithelial cells (26). Fourth, the expansion or functional shift of aging-related regulatory T cells, myeloid-derived suppressor cells and tumor-related macrophages may promote the formation of an inhibitory microenvironment supporting immune escape (27). Finally, inflammatory aging may provide a continuous source of cytokine signaling, promote matrix remodeling, tumor cell survival and activation of androgen receptor related pathways. These mechanisms suggest that immune aging may help explain why prostate cancer often shows weak anti-tumor immunity and persistent tumor supporting inflammation at the same time (28, 29) (**Figure 1**).

## 3 Aging-related immune dysfunction and prostate cancer progression

Aging-related immune dysfunction may affect prostate cancer progression at multiple levels, from the establishment of tumor-permissive niche to the weakening of effective anti-tumor immune surveillance (30, 31). Aging prostate is not only exposed to cumulative molecular damage, but also in a tissue environment characterized by chronic inflammation, matrix remodeling and changes in immune cell composition (32, 33). These age-related changes can create favorable conditions for malignant transformation by promoting epithelial cell instability, enhancing survival-promoting signaling and destroying the normal immune-mediated clearance of precancerous cells (31, 34). In this context, immune aging is particularly important because it affects both systemic immunity and local immune interactions in the prostate microenvironment (33, 34).

A major consequence of immune aging is the decline in the ability of cytotoxic immune cells to recognize and effectively clear tumor cells (33, 35). The function of CD8+ T cells decreases with age, while the diversity of T-cell receptors is limited and the expression of inhibitory receptors is increased, which may limit robust antitumor responses to prostate cancer-related antigens (36, 37). Similarly, the decline of cytotoxic activity of natural killer cells may also weaken their early immune control over transformed cells (36, 38). At the same time, aging is usually accompanied by the expansion of immunosuppressive cell populations, including regulatory T cells, myeloid-derived suppressor cells and tumor-associated macrophages, which can further weaken immune surveillance and support immune escape (37, 39). As a result, tumor cells may persist and evolve in a setting where immune pressure is insufficient for clearance yet sufficient to select for more resistant phenotypes (38, 39).

Prostate cancer is frequently described as an immunologically “cold” tumor, but this should not be interpreted as a complete absence of immune involvement (34, 40). By contrast, immunologically “hot” tumors are typically characterized by dense cytotoxic T-cell infiltration, high antigen presentation, and strong responsiveness to immune checkpoint blockade, whereas “cold” tumors such as prostate cancer show sparse or excluded immune infiltrates, limited antigen presentation, and correspondingly weaker responses to immunotherapy. Rather, the prostate tumor microenvironment often contains immune cells that are quantitatively limited, spatially excluded, functionally exhausted, or diverted toward protumor and immunosuppressive roles (40, 41). Immunosenescence may amplify this phenotype through several complementary mechanisms (22). Age-related loss of naïve T cells and reduced T-cell receptor diversity may limit the generation of new tumor-reactive clones, while impaired dendritic-cell function may weaken antigen presentation and T-cell priming (25). At the same time, reduced cytotoxic activity of CD8^+^ T cells and natural killer cells may diminish effective tumor cell killing. In parallel, inflammaging can promote the recruitment and activation of suppressive myeloid populations, including myeloid-derived suppressor cells and tumor-associated macrophages, which may further inhibit effector T-cell function (27, 42). Stromal remodeling associated with aging and senescence may also create physical and biochemical barriers that restrict immune-cell infiltration (43). Thus, in prostate cancer, immunosenescence may contribute not only to reduced immune surveillance but also to the formation of a suppressive, poorly inflamed, and therapeutically resistant tumor microenvironment (24).

Another important aspect is that aging-related immune dysfunction may also shape the therapeutic response (39, 44). Decreased immune fitness may limit the efficacy of immunotherapy, while chronic inflammation and inhibitory myeloid cell infiltration may weaken the ability of standard therapy to induce durable anti-tumor immunity (45, 46). In addition, androgen deprivation therapy, chemotherapy or radiotherapy may interact with the aging immune system in a complex way, sometimes enhancing antigen release, but may also aggravate immune exhaustion or inflammatory dysregulation (44, 46). Taken together, these observations suggest that immune aging is not a simple background feature of older patients with prostate cancer, but an active biological process, which may be involved in the formation of tumor occurrence, progression, immune escape and treatment response differences (30, 47) (**Table 1**).

**Table 1:** Aging-associated immune alterations, mechanisms, relevance to prostate cancer, and potential therapeutic implications.

Aging-associated immune alteration	Underlying mechanism	Relevance to prostate cancer	Potential therapeutic implication	Evidence status
Contraction of naïve T-cell pool and reduced TCR diversity	Thymic involution and accumulation of terminally differentiated T cells	May limit recognition of prostate tumor-associated antigens and contribute to weak adaptive immune surveillance	Biomarkers of immune age; strategies to enhance antigen presentation and T-cell priming	Supported by aging immunology; prostate cancer-specific validation still needed
Impaired CD8^+^ T-cell and NK-cell cytotoxicity	Reduced proliferative capacity, impaired cytokine production, increased inhibitory receptor expression	May weaken elimination of transformed prostate epithelial cells and support tumor persistence	Combination immunotherapy; reversal of exhaustion-like states; immune fitness assessment	Supported in cancer and aging studies; partially supported in prostate cancer
Defective dendritic-cell antigen presentation	Altered maturation, migration, and T-cell priming capacity	May reinforce the immunologically “cold” phenotype of prostate cancer	Dendritic-cell activation, vaccines, radiotherapy or targeted therapies that enhance antigen release	Biologically plausible; requires stronger clinical validation
Expansion of suppressive immune populations	Increased Treg cells, MDSCs, and tumor-associated macrophages in aged or inflamed tissues	May promote immune escape, suppress effector T cells, and limit response to immunotherapy	Targeting myeloid recruitment, macrophage polarization, or Treg-mediated suppression	Supported by tumor immunology; prostate cancer-specific mechanisms increasingly recognized
Inflammaging	Chronic low-grade IL-6, TNF-α, IL-1β and related inflammatory signaling	May promote tumor cell survival, stromal remodeling, androgen receptor signaling, and metastatic progression	Anti-inflammatory or cytokine-modulating strategies; patient stratification by inflammatory burden	Supported mechanistically; therapeutic translation remains under investigation
Cellular senescence and SASP	Accumulation of senescent tumor, stromal, epithelial, or therapy-exposed cells secreting inflammatory mediators	May amplify chronic inflammation, recruit suppressive myeloid cells, and promote resistance or relapse	Senolytic or senomorphic therapy combined with ADT, radiotherapy, or chemotherapy	Emerging evidence; largely preclinical or early translational
Stromal remodeling and immune exclusion	Aging- and SASP-associated extracellular matrix remodeling and fibroblast activation	May restrict immune infiltration and reinforce a suppressive tumor niche	Microenvironmental reprogramming; stromal-targeted combinations	Plausible and increasingly supported; requires further validation

TCR, T-cell receptor; NK, natural killer; Treg, regulatory T cell; MDSC, myeloid-derived suppressor cell; SASP, senescence-associated secretory phenotype; ADT, androgen deprivation therapy.

## 4 Cellular senescence, SASP, and immune remodeling in prostate cancer

In addition to immune aging, cellular senescence has become another important aging-related process in prostate cancer biology (48, 49). Cellular senescence refers to a stable cell cycle arrest state triggered by a variety of stress factors, including telomere shortening, oncogenic signaling, DNA damage, oxidative stress and anti-cancer treatment (49, 50). Although cell senescence can initially exert tumor inhibitory effect by preventing the continued proliferation of damaged cells, its long-term consequences are more complex (50, 51). Senescent cells still maintain metabolic activity and secrete a wide range of inflammatory cytokines, chemokines, growth factors and proteases, which together constitute the senescence-associated secretory phenotype (SASP) (51, 52). For example, SASP components such as interleukin-6, interleukin-8, and matrix metalloproteinases have been shown to promote angiogenesis, remodel the extracellular matrix, and recruit immunosuppressive myeloid cells, thereby contributing to tumor progression and therapy resistance in several cancer types. In prostate cancer, senescent cells may not only come from tumor cells, but also exist in stromal fibroblasts, epithelial cells and tissues exposed to treatment, thus affecting the local tumor microenvironment in a variety of ways (48, 53).

SASP provides an important mechanism for cell senescence and immune remodeling. On the one hand, SASP factors can recruit immune cells and promote the clearance of senescent or damaged cells, suggesting that it has a transient tumor limiting effect (52, 54). On the other hand, persistent SASP signaling may promote chronic inflammation, matrix activation, extracellular matrix remodeling, and the recruitment of immunosuppressive cell populations (54, 55). Cytokines such as interleukin-6, interleukin-8, CCL2 and transforming growth factor-β are particularly relevant in this process, because they cannot only strengthen tumor supporting inflammation, but also weaken effective anti-tumor immunity (52, 55). In aging hosts, the level of basic inflammation has increased, and continuous SASP production may further amplify inflammatory aging and aggravate immune dysfunction in prostate tumor niche (55, 56).

The interaction between cell senescence and immune senescence is particularly important in prostate cancer, because most patients are older, and the treatment received by many patients can induce cell senescence (53, 57). Androgen deprivation therapy, radiotherapy and chemotherapy may promote treatment-induced cell senescence and have double consequences (50, 57). On the one hand, inducing cell senescence can temporarily inhibit the proliferation of tumor cells; On the other hand, the persistence of senescent cells after treatment may promote recurrence, drug resistance and persistent immune dysfunction through SASP-driven signal transduction (57, 58). In addition, the aging immune system may be difficult to effectively remove senescent cells, allowing them to accumulate and prolong their harmful effects (56, 58). Therefore, in prostate cancer, cellular senescence should not be regarded as an isolated process, but as a process closely intertwined with immune aging, chronic inflammation and microenvironment remodeling. Together, these interconnected pathways may shape tumor progression and help explain why aging profoundly influences prostate cancer biology and therapeutic response (53, 58).

## 5 Therapeutic opportunities targeting immunosenescence

Although therapeutic targeting of immunosenescence in prostate cancer is an emerging concept, the strength of evidence varies across different strategies (22, 42). Some observations, such as age-associated impairment of T-cell function, increased inflammatory signaling, and the immunologically cold nature of many prostate tumors, are supported by substantial experimental and clinical evidence (7). In contrast, approaches such as immune-age-guided patient stratification, senolytic or senomorphic therapy, and rational combinations designed specifically to reverse aging-related immune dysfunction remain largely investigational (59). Therefore, the therapeutic implications discussed below should be interpreted according to their current level of validation, ranging from established biological rationale to forward-looking strategies that require further preclinical and clinical testing.

The growing recognition of immunosenescence as a contributor to prostate cancer progression has important therapeutic implications (60, 61). Because aging-related immune dysfunction affects antitumor surveillance, inflammatory signaling, and treatment responsiveness, therapeutic strategies should move beyond tumor-intrinsic targets alone and consider the biological age of the immune system (61, 62). This is particularly important in prostate cancer, because the disease usually occurs in elderly men with immune changes, and standard treatment may further reshape immune function (62, 63). Therefore, interventions aimed at improving immune capacity, alleviating chronic inflammation or reprogramming tumor microenvironment may enhance treatment benefits in specific patients (60, 63).

A major area of concern is the optimization of immunotherapy in the context of immune aging. Although the efficacy of immune checkpoint inhibitors in unselected prostate cancer population is limited, this may partly reflect the existence of “cold” and inhibitory microenvironment from an immunological perspective, rather than a complete lack of therapeutic potential (64, 65). The decline of T-cell diversity, cytotoxic function and antigen reactivity associated with aging may limit the ability of older patients to produce durable anti-tumor immunity after receiving checkpoint blocking therapy (61, 65). Therefore, future immunotherapy strategies may need to include biomarkers such as immune age, inflammatory state or aging load to identify patients who are more likely to benefit (62, 66). Combination regimens that enhance antigen presentation, increase T-cell infiltration, or reduce suppressive myeloid signaling may be especially important in overcoming age-related resistance mechanisms (64, 67).

A second promising strategy involves reprogramming the prostate tumor microenvironment. Since immunosenescence is accompanied by chronic inflammation and the accumulation of suppressive cell populations such as regulatory T cells, myeloid-derived suppressor cells, and tumor-associated macrophages, targeting these components may help restore more effective immune surveillance (63, 67). Agents that modulate inflammatory cytokines, inhibit protumor myeloid recruitment, or reverse macrophage polarization could potentially reduce the permissive conditions created by the aged microenvironment (61, 68). In parallel, therapies that promote dendritic-cell function or improve antigen presentation may strengthen the link between innate and adaptive immunity, thereby increasing the effectiveness of both conventional treatment and immunotherapy (66, 68).

Another emerging opportunity lies in targeting cellular senescence and the SASP. Because therapy-induced senescence may suppress proliferation in the short term but contribute to chronic inflammation, relapse, and immune dysfunction over time, strategies that eliminate senescent cells or attenuate their secretory phenotype have attracted growing attention (69, 70). Senolytic agents may help remove persistent senescent cells, whereas senomorphic approaches aim to suppress harmful SASP signaling without necessarily inducing cell death (70, 71). In prostate cancer, these approaches may be particularly attractive when combined with androgen deprivation, radiotherapy, or chemotherapy, where treatment-related senescence is likely to occur (69, 71). By reducing the inflammatory and immunosuppressive effects of aging cell accumulation, such interventions may improve local tumor control and the overall immune environment at the same time (70, 72).

In the final analysis, the treatment relevance of immune aging supports the management of prostate cancer from a more individualized perspective. Chronological age is unlikely to fully reflect the heterogeneity of immune aging between patients (62, 72). This discrepancy may be influenced by genetic background, chronic infections such as CMV, comorbidities, lifestyle factors, cumulative inflammatory exposure, and prior cancer therapies; potential biomarkers include DNA methylation or epigenetic clocks, telomere length, T-cell receptor diversity, naive/memory T-cell ratios, CD8+ T-cell senescence markers, p16INK4a expression, circulating inflammatory mediators such as IL-6, TNF-alpha and CRP, and SASP-related factors. Instead, the integration of markers related to immune function, inflammatory load and cellular senescence may help to more accurately classify patients and guide reasonable combined treatment strategies. A better understanding of how immunosenescence interacts with tumor biology may therefore open new avenues for improving outcomes in older men with prostate cancer (60, 72) (**Table 2**).

**Table 2:** Therapeutic opportunities targeting immunosenescence in prostate cancer.

Therapeutic direction	Biological rationale	Representative strategies	Potential significance
Overall therapeutic implication	Aging-related immune dysfunction weakens antitumor surveillance, sustains chronic inflammatory signaling, and alters treatment responsiveness.	Design strategies beyond tumor-intrinsic targets; incorporate biological age of the immune system into treatment planning.	Provides a broader framework for therapy selection in older patients with prostate cancer.
Optimization of immunotherapy	Reduced T-cell diversity, cytotoxicity, and antigen responsiveness may limit durable benefit from checkpoint blockade in immunologically ‘cold’ prostate cancer.	Use biomarkers of immune age, inflammatory status, or senescence burden; combine checkpoint inhibitors with approaches that enhance antigen presentation and T-cell infiltration or reduce myeloid suppression.	May improve patient selection and help overcome age-related resistance to immunotherapy.
Reprogramming the tumor microenvironment	Immunosenescence is associated with chronic inflammation and accumulation of suppressive cell populations, including Treg cells, MDSCs, and tumor-associated macrophages.	Modulate inflammatory cytokines, inhibit protumor myeloid recruitment, reverse macrophage polarization, and improve dendritic-cell function and antigen presentation.	May restore more effective immune surveillance and increase the efficacy of conventional therapy and immunotherapy.
Targeting cellular senescence and SASP	Therapy-induced senescence may temporarily suppress proliferation but later promote chronic inflammation, relapse, and immune dysfunction through SASP signaling.	Apply senolytic agents to eliminate persistent senescent cells or senomorphic approaches to attenuate harmful SASP without inducing cell death; combine with ADT, radiotherapy, or chemotherapy.	May reduce inflammatory and immunosuppressive consequences of senescent-cell accumulation and improve local as well as systemic control.
Personalized management based on immune aging	Chronological age alone does not capture heterogeneity in immune aging among patients.	Integrate markers of immune function, inflammatory burden, and senescence into patient stratification and rational combination therapy design.	Supports more precise and individualized management in older men with prostate cancer.

## 6 Discussion and future directions

Immunosenescence provides a valuable framework for understanding why prostate cancer is strongly associated with aging and why disease behavior may differ substantially among older patients. Current evidence supports the concept that aging is accompanied by reduced antitumor surveillance, impaired effector-cell function, contraction of the naive T-cell compartment, chronic low-grade inflammation, and increased immunosuppressive remodeling. These alterations are biologically relevant to prostate cancer because the disease commonly arises in older men and frequently exhibits an immunologically cold and suppressive tumor microenvironment. Evidence from aging immunology, tumor immunology, and prostate cancer studies collectively suggests that immunosenescence may contribute to impaired tumor antigen recognition, reduced cytotoxic immune activity, suppressive myeloid infiltration, stromal remodeling, and inflammatory signaling that favors tumor persistence and progression.

At the same time, several aspects of the framework have not been fully verified in prostate cancer. For example, although the general characteristics of immune aging have been relatively clear, the exact role of specific immune aging cell populations in the occurrence, metastasis and progression of prostate cancer and treatment resistance still need to be further studied. Similarly, the interaction between androgen deprivation therapy, radiotherapy, chemotherapy and other standard prostate cancer treatment and aging immune or matrix microenvironment is still complex. Under certain conditions, these therapies may promote antigen release and immune activation, but may also induce cell senescence, SASP production, immune exhaustion or chronic inflammatory remodeling. More mechanism studies and prospective clinical analysis are still needed to determine when these effects have therapeutic benefits and when they may promote drug resistance or recurrence.

From the perspective of translational medicine, the existing evidence supports that chronological age is no longer the only clinical index to describe the older patients with prostate cancer. However, it is still an important but developing strategy to use immune age, inflammatory load or aging-related biomarkers for treatment selection. Future research should focus on identifying robust and clinically useful biomarkers of immune aging, clarify how these biomarkers are related to tumor immunophenotypes, and determine whether they can predict patients’ response to immunotherapy, androgen receptor targeted therapy, radiotherapy or combination therapy.

Some therapeutic directions are promising, but they should still be regarded as prospective strategies. These directions include reprogramming the tumor microenvironment of aging, enhancing antigen presentation, reversing inhibitory myeloid signaling, regulating inflammatory aging, and targeting cellular senescence or SASP. Senolytic and senomorphic strategies are particularly attractive because the treatment of induced aging may promote chronic inflammation and immunosuppression; However, its clinical value in prostate cancer remains to be established. Similarly, the combined treatment strategy of enhancing anti-tumor immunity and limiting chronic inflammation and immunosuppressive signals also needs to be carefully verified to avoid excessive toxicity or accidental immune imbalance.

In conclusion, the more precise integration of tumor immunology and aging biology may help to deepen the understanding of the mechanism of prostate cancer and optimize treatment decisions. Well-supported evidence indicates that immune aging can alter anti-tumor surveillance and inflammatory remodeling, while emerging hypotheses suggest that targeting these aging-related immune and stromal changes may improve therapeutic outcomes. Distinguishing between these evidence levels will be essential for developing rational, biologically informed strategies for older men with prostate cancer.
